# Special Issue: Arts Therapies with Children and Adolescents—Editorial

**DOI:** 10.3390/children10010110

**Published:** 2023-01-04

**Authors:** Dafna Regev

**Affiliations:** 1 The School of Creative Arts Therapies, University of Haifa, Haifa 3498838, Israel; daf.regev@gmail.com; 2 The Emili Sagol Creative Arts Therapies Research Center, University of Haifa, Haifa 349838, Israel

Arts therapy dates back to the mid-20th century. It emerged from the conviction that artistic work has a unique meaning for people in general and children and adolescents in particular. Although many professionals use the arts in their work with children and adolescents, arts therapists have specific expertise in observing and encouraging processes in a variety of arts and have the knowledge base necessary to promote connections between artistic creation and stimulate mental processes and personal well-being.

Clinical work in arts therapy is expanding to education, hospitals, informal education, private clinics and other settings. Similarly, the research field has developed rapidly, especially in the last twenty years, and today includes a growing number of in-depth studies that not only examine the effectiveness and meaning of this profession, but also explore therapeutic processes and mechanisms of change and contribute to the formulation of protocols adapted to therapy work in a variety of populations. While many studies have focused on adults in arts therapy, research on arts therapy for children and adolescents still lags behind. This points to the need to find specific ways to treat these clients and for studies on how these approaches can be implemented.

For all these reasons, I am delighted to serve as the guest editor representing the field of arts therapy for this Special Issue in *Children*. This Special Issue presents a wide range of articles. First and foremost, several deal with arts therapy in the education system. Heynen and her associates [1] present a specific music therapy intervention developed in the Netherlands for refugee children and adolescents in school settings. Snir [2] explores the meaning of artmaking as one of the key components of art therapy within the educational system in Israel. Kelemen and Shamri-Zeevi [3] describe a unique open-studio intervention designed to facilitate identity development in teens recovering from mental health conditions. Korman-Hacohen and her collaborators [4] specifically refer to the challenges brought about by the COVID-19 pandemic and the creative way in which arts therapists in the education system continued to work with students by harnessing new and different approaches.

The second topic discussed in this Special Issue is arts therapies for children and adolescents with special needs. Bat-Or and Zusman-Bloch [5] describe art therapy in an open-studio model with at-risk children living in foster care. Schweizer and her colleagues [6] report on a 15-session art therapy program that aims to reduce difficulties in ‘sense of self’, ‘emotion regulation’, ‘flexibility’ and ‘social behavior’ in children diagnosed with autism spectrum disorders (ASDs). Bitan and Regev [7] investigate ways to work with clients with ASDs through parent–child art psychotherapy. Cousin and collaborators [8] describe music therapy interventions in pediatric intensive care units for anxiety and pain management. Ofer and Keisari [9] present a case study and the core concepts implemented during drama therapy with a young girl who lost most of her functional abilities due to brain damage. During the child’s physiotherapy sessions at the rehabilitation hospital, a medical clown was brought in to work together with the physiotherapist in providing treatment. 

Beyond the therapeutic use of the various arts, greater attention is being paid to the diagnostic potential of arts therapies. These diagnostic methods are grounded in the realization that speech is not always the most appropriate channel for diagnosis, especially in children and adolescents. Bat-Or and her partners [10] describe diagnosis based on the Person Picking an Apple from a Tree (PPAT) drawing assessment scale. They evaluated the subjective experience of 156 preschoolers (aged 4–6.9 years) living in an area exposed to considerable political violence in Israel (on the border with the Gaza Strip) during a period of massive bombing. Gavron and her partners [11] describe a painting intervention called the Joint Painting Procedure (JPG) where parent and child paint together on the same sheet of paper. This is used to examine key facets of the relationships between adolescents with intellectual disabilities and their mothers. Jaroenkajornkij and her associates [12] provide a new look at the classic self-figure drawing, which they use to successfully identify three forms of child abuse: child sexual abuse, child physical abuse and child emotional abuse.

The last section deals with more general issues in the field of arts therapy for children and adolescents. Shuper-Engelhard and Vulcan [13] examine the distinctive qualities of group dance and movement therapy in the context of a remote emotional intervention with young children. Metzl [14] reviews current theoretical frameworks of working with children and adolescents with regard to their socio-political and developmental implications for art therapy practice within different settings and systems. The systematic review by Berghs and her associates [15] looks at the ways in which drama therapy contributes to a decrease in psychosocial problems. Moula and collaborators [16] conducted a pilot randomized controlled study that examines the effects of arts therapies on children’s mental health and well-being. Keidar and her associates [17] explore the perceptions of 17 ultra-Orthodox parents whose children were receiving arts therapies. 

I hope that this Special Issue will serve as a repository of knowledge for arts therapists and fertile terrain for further research in the field. It also aims to help more professionals working with children and adolescents to recognize the meaning and uniqueness of therapeutic work in arts therapies and the dedicated ways in which arts therapists use assessment tools and arts-based interventions to better understand the world of children and adolescents.
